# Local lockdowns outperform global lockdown on the far side of the COVID-19 epidemic curve

**DOI:** 10.1073/pnas.2014385117

**Published:** 2020-09-04

**Authors:** Vadim A. Karatayev, Madhur Anand, Chris T. Bauch

**Affiliations:** ^a^School of Environmental Sciences, University of Guelph, Guelph, ON N1G 2W1, Canada;; ^b^Department of Applied Mathematics, University of Waterloo, Waterloo, ON N2L 3G1, Canada

**Keywords:** COVID-19, pandemic mitigation, spatially structured model, stochastic model, epidemic model

## Abstract

In the late stages of an epidemic, infections are often sporadic and geographically distributed. Spatially structured stochastic models can capture these important features of disease dynamics, thereby allowing a broader exploration of interventions. Here we develop a stochastic model of severe acute respiratory syndrome coronavirus 2 (SARS-CoV-2) transmission among an interconnected group of population centers representing counties, municipalities, and districts (collectively, “counties”). The model is parameterized with demographic, epidemiological, testing, and travel data from Ontario, Canada. We explore the effects of different control strategies after the epidemic curve has been flattened. We compare a local strategy of reopening (and reclosing, as needed) schools and workplaces county by county, according to triggers for county-specific infection prevalence, to a global strategy of province-wide reopening and reclosing, according to triggers for province-wide infection prevalence. For trigger levels that result in the same number of COVID-19 cases between the two strategies, the local strategy causes significantly fewer person-days of closure, even under high intercounty travel scenarios. However, both cases and person-days lost to closure rise when county triggers are not coordinated and when testing rates vary among counties. Finally, we show that local strategies can also do better in the early epidemic stage, but only if testing rates are high and the trigger prevalence is low. Our results suggest that pandemic planning for the far side of the COVID-19 epidemic curve should consider local strategies for reopening and reclosing.

## Significance

During the COVID-19 pandemic, decision makers are grappling with how to reopen (and possibly reclose) their jurisdictions as the number of cases ebbs and flows. Establishing a criterion for each county/municipality to open and close based on their case count has appeal, given the wide disparity in COVID-19 rates in urban versus rural settings. Our simulation model is based on the geography, epidemiology, and travel patterns of Ontario, Canada. It shows that the county-by-county approach causes fewer days of closure and impacts fewer people than a strategy that opens or closes the entire province together. This is true even if individuals begin traveling to reopened counties with higher frequency. The county-by-county strategy is most effective when the criteria are coordinated.

Outbreak containment through testing, case isolation, contact tracing, and quarantine is often the first line of defense against a novel emerging infectious disease (1–3). However, efforts to contain severe acute respiratory syndrome coronavirus 2 (SARS-CoV-2) outbreaks have failed in many jurisdictions, including Ontario, Canada, leading decision makers to supplement contact tracing with effective but socioeconomically costly interventions such as school and workplace closure and other means of physical distancing (4, 5).

These measures have flattened the epidemic curve: They have reduced the effective reproduction number of SARS-CoV-2 below one, meaning that each infected case is infecting less than one person on average (5). The epidemic curve is a common way to visualize the spread of an infectious disease and has become ubiquitous during the COVID-19 pandemic. Data on cases over time lend themselves naturally to analysis by compartmental epidemic models that assume a homogeneously mixing population. Such models can be a valid approximation for many applications (6, 7). However, the epidemic curve can also obscure the spatiotemporal nature of infectious diseases, as infections jump between neighboring populations (8). In the early stages of an outbreak, cases are few and thus subject to random effects (stochasticity). And, in the late stages of an outbreak, cases are both stochastic and spatially dispersed across multiple population centers connected through travel (9).

In such early and late stages of an epidemic, a stochastic, spatially structured model can capture important features of disease dynamics (10–13). When cases are rare, the infection may go locally extinct due to chance events—an effect referred to as stochastic fade-out (8, 14, 15). This has nontrivial interactions with the spatial structure of the population (16). If cases are still high in other populations, the virus may be subsequently reintroduced from those other populations through travel (8, 17). But, if the infection has also faded out in the other populations, the virus is eradicated (18).

As cases continue to decline on the far side of the COVID-19 epidemic curve in Ontario, decision makers will make choices about how and when to lift restrictions. But they will face a very different epidemiological landscape than the middle stages of the outbreak, when infections were numerous. Complete and sudden removal of these restrictions before a sufficient proportion of the population is immune to SARS-CoV-2 could cause a resurgence of cases (19). Hence a phased approach to open or close schools and workplaces, based on “trigger” conditions such as the number of local confirmed positive cases, might be better (4, 19).

**Fig. 1.**
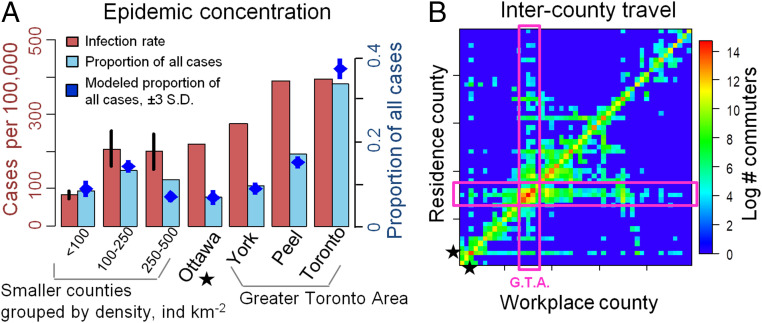
Concentration of COVID-19 cases in (*A*) urban counties and (*B*) travel patterns among counties. Bars in *A* denote infection rate (cumulative cases per 100,000; red) and proportion of all cases in Ontario (blue) across counties by June 10, 2020 (26), and blue points denote the fit of 20 stochastic model realizations. Counties with <1 million residents are aggregated by population density (individuals per square kilometer), with vertical lines denoting standard error across counties. In *B*, boxes denoted G.T.A. represent the Greater Toronto Area, and star denotes Ottawa; data are from ref. 37.

Phased approaches might be temporal in nature, with certain types of workplaces being opened before other types, for instance. Alternatively, a spatially phased approach is also possible, with smaller and/or less densely populated areas being reopened before larger urban centers (20). Spatially phased approaches are based on the hypothesis that, during the later stages of a pandemic, the force of infection in smaller populations could be significantly less than larger populations, due to more frequent stochastic fade-out (8, 14, 15), reduced contact rates on account of lower population densities (21–24), and/or reduced case importation due to fewer travel connections (25). Indeed, Ontario’s four largest cities have 2.5 times more COVID-19 cases per capita than the rest of the province (Fig. 1*A*) (26). Hence, school and workplace closure could be lifted first in those populations where they provide little marginal benefit. But, under a spatially phased approach, coordination remains paramount (27), given that pathogens can spread rapidly between populations during a pandemic (4, 25). Reimportation risk may compound when local closures are poorly coordinated, with some populations eager to lift closures and hesitant to reenact them when needed.

These observations emphasize three things about modeling COVID-19 interventions on the far side of a flattened curve: 1) A stochastic transmission model might be useful for capturing disease dynamics once cases become rare; 2) spatial structure is important for evaluating spatially phased plans, since the pathogen will not be present everywhere all of the time; and 3) cases can be reimported from other locations that have not eliminated the infection, suggesting that coordination between counties under a spatially phased approach could remain important. Our objective is to develop a spatially structured stochastic model of SARS-CoV-2 transmission, testing, and school and workplace closure in Ontario in order to address three questions: 1) Are closures best lifted at the scale of an entire province or on a county-by-county basis? 2) Does coordination of testing protocols and reopening criteria between counties improve outcomes? 3) How well can a spatially phased approach work in the early stages of the epidemic? We use our model to determine the timing and organizational scale at which school and workplace reopening strategies can minimize both the number of infections and person-days lost to closures, during the late-stage and early-stage epidemic. Our model is parameterized with epidemiological, demographic, and travel data for the counties, municipalities, and districts (collectively, “counties”) of Ontario, Canada.

## Results

### Model Overview.

We model a population distributed across local population centers (“counties”) connected through travel. Within each county, transmission follows a SEPAIR disease natural history: S is susceptible to infection, E is infected, but not yet infectious (or, simply, “exposed”), P is presymptomatic infectiousness (or, simply, “presymptomatic”), A is infectious without ever developing symptoms (or, simply, “asymptomatic”), I is both infectious and symptomatic (or, simply, “symptomatic”), and R is removed (no longer infectious). Symptomatic individuals are tested for SARS-CoV-2, and their status becomes ascertained with some probability per day. The infection transmission probability in a county depends on the number of contacts in schools and workplaces—which are reduced by closures—and on contacts in other settings not affected by closures, such as homes. Transmission also depends on how effectively closures reduce transmission, and the extent to which population size drives transmission. The population behavioral response to the presence of COVID-19 is an important feature of physical distancing (28–32). Hence, we assumed that transmission outside of schools and workplaces is reduced by individual physical distancing efforts (restricting social contacts, washing hands, etc.) and that more confirmed positive cases in the county cause more individuals to practice physical distancing. Each individual travels from their home county to another county for the day with some probability (Fig. 1*B*) that is reduced if schools and workplaces are closed in the destination county. Additional details on model structure, data sources, parameter values, and calibration appear in *Materials and Methods*. Parameter definitions, values, and literature sources are summarized in *SI Appendix*, Table 1.

### System Dynamics.

**Fig. 2.**
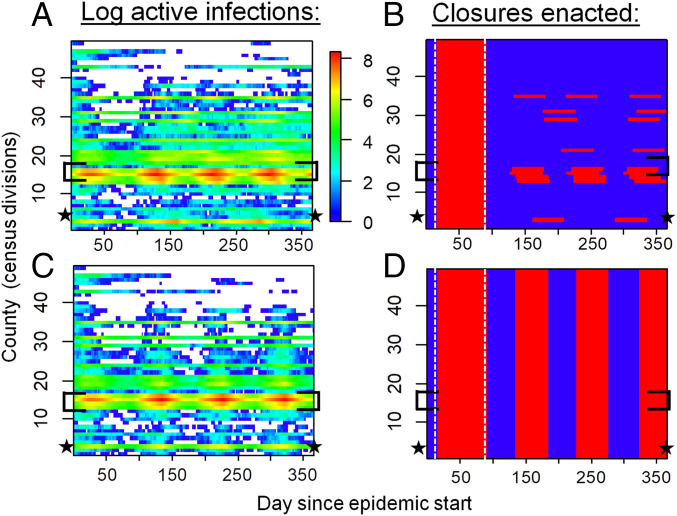
Spatiotemporal patterns of local COVID-19 cases and school/workplace closures. (*A* and *C*) Simulated (confirmed plus not ascertained) number of COVID-19 cases and (*B* and *D*) time periods of workplace closure (red) and opening (blue) in Ontario, Canada under (*A* and *B*) local and (*C* and *D*) global strategies. Disease dynamics are sporadic in low-population counties but endemic in high-population counties. The local strategy generates a similar disease burden to the global strategy but requires fewer person-days of closure. Optimal trigger prevalence is assumed (blue dashed lines, Fig. 3). Brackets denote the Greater Toronto Area, star denotes Ottawa, and vertical dashed lines in *B* and *D* delineate the initial province-wide closure. All simulations were initialized with 2,500 exposed persons on day 1.

We ran reopening and reclosing simulations over a time horizon of 1 y and projected the number of cases in each county. To reflect COVID-19 mitigation in Ontario, each simulation began with a 75-d period of province-wide closure applied once 325 confirmed positive cases accumulated in the province. After this period, we contrasted a “local strategy” of reopening and reclosing counties individually, according to a trigger prevalence of confirmed positive COVID-19 cases in the county, to a “global strategy” of reopening and reclosing the entire province, according to a trigger prevalence of confirmed positive COVID-19 cases in the province. Our model dynamics are characterized by two distinct regimes (Fig. 2 *A* and *C*). In highly populated counties, COVID-19 is endemic throughout the time horizon of the simulation. However, in counties with lower populations, cases blink in and out during the year, as infections jump between counties through travel and decline due to testing and voluntary distancing alone. The infection patterns appear qualitatively similar under both strategies (Fig. 2 *A* and *C*), but closure patterns are very different, with most counties being closed most of the time under the global strategy (Fig. 2 *B* and *D*).

### Local versus Global Reopening Strategies.

**Fig. 3.**
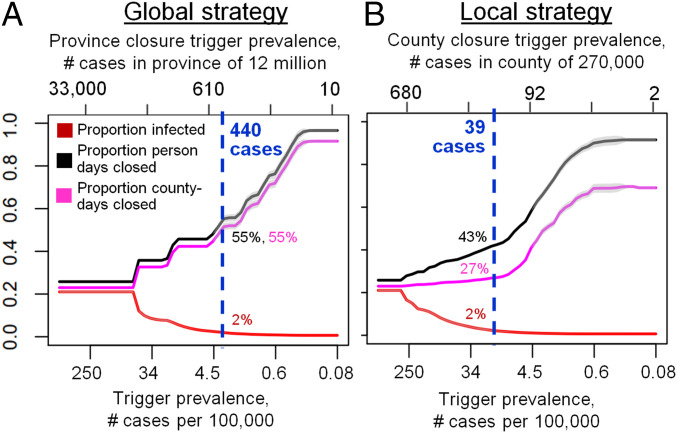
The local strategy greatly reduces person-days of school and workplace closure while only causing a small increase in the number of COVID-19 cases, relative to the global strategy. Effect of trigger prevalence on proportion infected (red) and proportion of person-days under closure (black) for the (*A*) global and (*B*) local strategy. Vertical blue lines denote optimal trigger prevalence that maintains proportion infected within 1% of its minimum value while minimizing person days closed. Shading represents ±2 standard deviations across 30 model realizations.

The local strategy tends to outperform the global strategy for most values of the trigger prevalence (Fig. 3). When the trigger prevalence is very high (i.e., an extreme scenario where decision makers reopen or reclose for a prevalence of 1,000 confirmed positive cases per 100,000), a high proportion of the population becomes infected, since school and workplace closures are rarely sustained in either strategy after the initial 75-d province closure. At the other extreme of the lowest trigger prevalence, both strategies minimize infections by maintaining closures for the majority of the year. However, intermediate values of the trigger prevalence represent a “sweet spot” for the local strategy, where it outperforms the global strategy in terms exhibiting significantly fewer person-days of closure for a comparable number of COVID-19 cases. The local strategy can accomplish this because it affords flexibility to enact closures only in areas with continuing active outbreaks—primarily, more populous counties with higher epidemic spread rates. Conversely, for the same trigger prevalence, cases of infection in this regime are always lower under the local strategy. We identify an optimal trigger prevalence as the trigger prevalence that allows significant reductions in person-days lost to closure, but only permits cases to increase by 1% compared to its minimum value across all values of the trigger prevalence (blue dashed lines, Fig. 3). At this optimal trigger, the local strategy results in 22% fewer person-days of closure across the entire province than the global strategy.

### Benefits of Coordination.

**Fig. 4.**
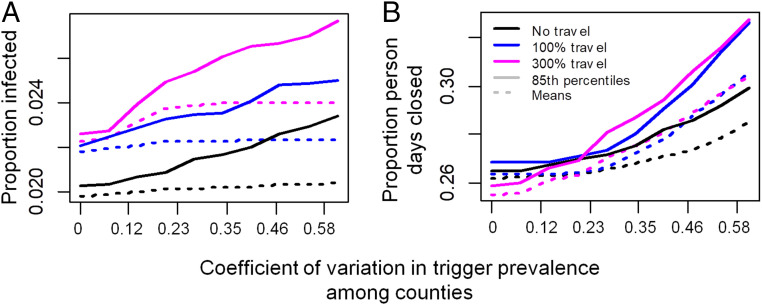
Decreasing coordination in trigger prevalence across counties increases (*A*) cases and (*B*) person-days closed under the local strategy. $\gamma_{l,j}$ follows uncorrelated variation among counties according to a uniform distribution with mean $\gamma_{l}=15$ cases/100,000 at the optimal trigger prevalence in Fig. 3*B*.

A local strategy could enable different counties to adopt different triggers. Our simulation results confirm that poor coordination can undermine the benefits of the local strategy (Fig. 4). As between-county variation in the trigger prevalence increases, both the mean and 85% quantile across stochastic realizations of both the proportion infected and person-days closed rise under a broad range of assumptions for intercounty travel rates (Fig. 4). The rise in infections in this scenario is somewhat counteracted by the rise in person-days lost to closure: Renewed outbreaks in counties that lift closures prematurely export infections to neighboring counties, which, in turn, necessitates additional closures in those counties and increases the number of person-days lost to closure (Fig. 4*B*). This emphasizes how close coordination can be beneficial from both public health and economic perspectives. Lack of coordination in testing is also problematic (*SI Appendix*, Fig. 1). As between-county variation in the testing rate for symptomatic individuals increases, the mean and 85% quantile of proportion infected and person-days lost to closure increase in most of the stochastic realizations.

### Sensitivity Analysis.

These results are qualitatively unchanged under moderate changes to parameter values in univariate sensitivity analyses (*SI Appendix*, Fig. 2). Projections are most sensitive to variation in the transmission probability, efficacy of physical distancing, and the removal rate. The performance of the local versus global strategies depends relatively little on the extent to which transmission probabilities are driven by population size, in other words, how rapidly the probability that a given susceptible person is infected by a given infectious person declines with the population size of the county (ξ is changed and model is refitted with new ξ values; *SI Appendix*, Fig. 3). Similarly, the relative performance of the two strategies is not strongly affected by doubling of travel rates (*SI Appendix*, Fig. 4): Although cases and person-days lost to closure increase for both strategies, the local strategy retains its relative performance lead over the global strategy.

### Local Closures in the Early Epidemic.

**Fig. 5.**
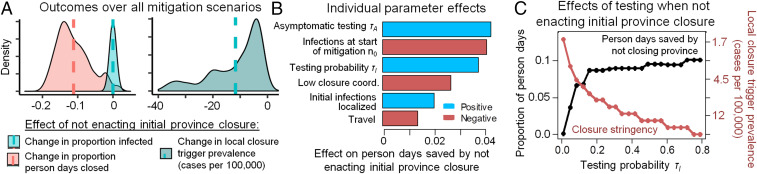
Using county-by-county closures from the beginning and omitting the initial 75-d province-wide closure at the start of an epidemic can minimize infections and person-days of closure under moderate-to-high testing rates and low trigger prevalence. (*A*) Across all combinations of control parameters (specified in *B*), not enacting an initial 75-d province closure at the start of the epidemic reduces person-days closed but requires a lower trigger prevalence in county-level closure decisions (vertical lines denote means). (*B*) Sensitivity analysis of how each control parameter affects person-days of closure avoided by omitting the initial 75-d province-wide closure. (*C*) Moderate-to-high testing levels allow less stringent county closure criteria (trigger prevalence, red) and result in fewer person-days closed (black) compared to 75-d province-wide closure. Person-days lost, infections, and trigger prevalence were calculated over the first 120 d of the epidemic. Throughout this analysis, we use a baseline $\tau_{I}=0.16$. In *A* and *B*, $\tau_{I}$ and $n_{0}$ were varied over ±75% of their baseline values, low coordination is either absent or present (in which case, $\gamma_{l,j}$ among counties follows a uniform distribution with coefficient of variation 0.27), asymptomatic testing $\tau_{A}=0$ or $\tau_{A}=\tau_{I}/2=0.08$, and initial infections are either distributed evenly among the population or concentrated in two randomly selected counties which have a population of >500,000.

We also compared a modified local strategy of omitting the initial 75-d province-wide closure and closing counties one at a time from the very beginning (followed by reopening and reclosing counties as needed) to our baseline local strategy of following a 75-d province-wide closure with reopening and reclosing counties one at a time. We found that the modified local strategy could outperform the baseline local strategy under specific conditions for trigger prevalence and testing rates (Fig. 5). In particular, the trigger prevalence must be reduced compared to our baseline analysis (Fig. 5*A*), such that counties are closed as soon as a few cases are detected (Fig. 5*C*). The optimal trigger prevalence for the modified local strategy increases exponentially with the testing probability (from 17 to 120 positive active cases in a city the size of Ottawa), meaning that counties can apply less stringent triggers only if their testing rates are very high and find more cases (Fig. 5*C*). Testing of asymptomatic individuals is not included in our baseline analysis, but might occur under high testing capacity and effective contact tracing, and would permit a higher trigger prevalence and reduced person-days closed under the modified local strategy (Fig. 5*B*), by limiting epidemic growth to the most populous counties. However, given the initial short supply of test kits and long testing turnaround times that characterized Ontario and many other jurisdictions, the testing rate for symptomatic individuals probably remained below the required 0.1/d during the early epidemic in Ontario. Sensitivity analysis in the early epidemic (Fig. 5*B*) additionally shows that the benefit of fewer person-days closed under the local strategy declines when closures begin after many thousands of people are already infected, when travel is high, or if initial infections are concentrated in cities. Taken together, these results suggest that the modified local strategy of omitting the 75-d province-wide closure could significantly outperform the baseline local strategy early in the initial epidemic only with prompt mitigation, moderate-to-high testing rates, and very low trigger prevalence (a scenario resembling the South Korean control strategy). This finding reiterates public health consensus that early and aggressive action in the early stages of a pandemic, and also potentially during second waves, could minimize both infections and total person-days of closure.

## Discussion

Plans for reopening and reclosing schools and workplaces in the later stages of COVID-19 epidemics are diverse and uncoordinated. Some reopening guidelines include epidemiological triggers such as case incidence or contact tracing capacity (33), while others include guidelines for reopening on a county-by-county basis (20). Our results suggest that plans for reopening economies on the far side of the COVID-19 epidemic curve should consider preceding larger-scale reopenings with local reopenings. However, for this to work, the trigger conditions need to be coordinated by the province: Individual counties cannot draw up guidelines independently.

Our model was parameterized for the province of Ontario, Canada. Sensitivity analysis showed our results were robust to assumptions regarding transmission processes and travel patterns. The robustness of these results stems from being able to reopen more-sparsely populated counties that have a lower case burden and can benefit from stochastic fade-out more often than densely populated urban centers (Fig. 2). In turn, this suggests that the results may apply more broadly to other Canadian provinces and US states with similarly low population density and dispersed spatial structure. However, this would need to be confirmed with model extensions that are tailored to these other jurisdictions. Additionally, not all US states began their control efforts with a period of closure that was effective enough to flatten their epidemic curves, which is a crucial difference.

Province- and state-wide lockdowns have generated resistance from populations that feel the restrictions should not apply to them. This outcome is tied up with the phenomenon of policy resistance, where the nonlinear behavioral response to an intervention partly undermines the intervention (34). Nonlinear interaction between human and natural systems is pervasive (35), and epidemiological systems are no exception (31, 36). Behavioral feedback during the COVID-19 pandemic has manifested through 1) physical distancing to minimize individual infection risk in response to rising case reports (29) and 2) pushback against lockdowns on account of economic impacts or perceived restrictions on civil liberties. An evidence-informed and coordinated approach to lifting lockdowns in less densely populated areas first, such as the one we propose, might have the added benefit of improving compliance to measures in populations that perceive province-wide closure to cover a needlessly large section of the population. In a related vein, local closures may be more effective if local decision makers can enact closures more promptly than what is possible under a province-wide decision-making process.

Our model did not include several features that could influence predictions. A key assumption was that individual counties enact closures as soon as positive cases exceed a trigger prevalence. In practice, a delay could allow case notifications to surge. Hesitation to reclose counties as needed can be especially problematic because 1) an increase in deaths follows weeks behind an increase in cases and 2) the optimal trigger prevalence found here translates to closing counties when only a few active cases are detected (Fig. 3*B*). Future iterations of this model could include other important features such as age structure (19) or intensive care unit (ICU) capacity. These features could enable projecting the effect of reopening only primary and elementary schools, or using local ICU occupancy as a trigger. Future work could also explore the role of extraprovincial case importations in the late stage of the pandemic, which could become important once local cases become rare (8).

Data on SARS-CoV-2 epidemiology, interventions, and treatments will become both increasingly available and increasingly reliable as the COVID-19 pandemic unfolds. There is a corresponding urgent need to develop more-detailed models that can address a broader range of policy questions, so that evidence-based policy-making has more information upon which to base decisions. Stochastic, spatially structured models may become increasingly useful for informing reopening and reclosing strategies in the COVID-19 pandemic, by allowing decision makers to explore the potential advantages of coordinated county-by-county strategies.

## Materials and Methods

The following subsections describe the details of the model structure and parameterization. A table of parameter definitions, baseline values, and literature sources appears in *SI Appendix*, Table S1.

### Population Structure and Travel.

The model contains 49 local populations (“counties”) that represent each of 49 Ontario census divisions and have the same corresponding population sizes (37). At the start of each day, each individual in county j travels to county k for the day with probability $m_{jk}$, in which case they experience any transmission events that occur there. At the end of the day, they return back to county j. The values of the travel matrix $M=\left[m_{jk}\right]$ were obtained from survey data (37) spanning 39% of Ontario inhabitants, of whom 25% worked outside their census division, arriving at an aggregate daily travel probability of 10% per day. This likely overestimates the impact of travel, since we are treating each individual in the population as equally likely to travel to another county each day, whereas, in real populations, the same individuals tend to travel each day and tend to repeat their contacts with the same individuals. Infected individuals are less likely to travel by a factor r=0.19 (i.e., 19% less likely to travel), since 81% of reported COVID-19 cases are mild (38). Individuals who test positive are less likely to travel by a factor η=0.8 (39, 40), with travel by individuals sick and confirmed positive reduced by (1−η)(1−r). Additionally, each individual’s travel probability to a closed county is reduced by a factor ϵ, since fewer individuals travel to a county when its schools and workplaces are closed.

### Transmission and Testing.

The state $\left\{D_{i},T_{i}\right\}$ of individual i reflects both their epidemiological status $D_{i}\in \left\{S,E,A,P,I,R\right\}$ and their testing status, where $T_{i}\in \left\{N,K\right\}$ for not known/known infection status, respectively; $\left\{\cdot ,T_{i}\right\}$ denotes an individual with testing status $T_{i}$ and any of the five epidemiological states, with similar interpretation for $\left\{D_{i},\cdot \right\}$. $P_{j}$ denotes the population size of county j, and $P_{j}^{DT}$ denotes the number of individuals of state {D,T} in county j. Each time step lasts 1 d. During each day, each individual’s epidemiological status in county j is updated as follows: 1) Individuals in the {S,⋅} state become exposed with probability $\lambda_{j}$, entering the {E,⋅} state; 2) individuals in the {E,⋅} state become presymptomatic with probability (1−π)α and enter the {P,⋅} state, or become asymptomatic with probability πα and enter the {A,⋅} state; 3) individuals in the {P,⋅} state become symptomatic with probability σ, entering the {I,⋅} state; 4) individuals in the {I,N} state are tested with probability $\tau_{I,j}$, entering the {I,K} states; and 5) individuals in the {I,⋅} and {A,⋅} states are removed (are no longer infectious) with probability ρ, entering the {R,⋅} state. Infection history parameter values are obtained from epidemiological literature (41, 42). We assign each newly infected individual to be a superspreader with probability s=0.2 (43) and denote superspreading (nonsuperspreading ) individuals with the subscript s (e.g., $A_{s}$, $I_{s}$) (ns, respectively). Superspreaders infect others with a probability that is (1−s)/s times higher than nonsuperspreaders. Daily testing rates improved over the first 40 d of province-wide closure as testing increased 13-fold (44). Hence we assumed the daily testing probability in the model increased from $\tau_{I}^{t_{0}}$ to $\tau_{I}^{t_{f}}$ by day 40 after the 325th positive case was detected, in direct proportion to the smoothed increase in the daily number of tests from Ontario testing data. Physical distancing through closures or behavioral changes can reduce the probability of transmission by preventing up to a fraction ϵ of all contacts. Closure $C_{j}\left(t\right)$ (see below) of schools and workplaces in county j can be applied and lifted over time and affect a fraction w of all contacts. We take w=0.45 based on time use data for time spent at schools, workplaces, and other institutions that can be mandated to close (45). The remaining time spent, 1−w, is in settings such as homes and social gatherings. We assumed that individuals reduce their contacts in linear proportion to the number of confirmed cases reported in their county by a factor $1-\exp\left(-\omega P_{j}^{+}/P_{j}\right)$, where $P_{j}^{+}=P_{j}^{AK}+P_{j}^{IK}$. Hence, the fraction of contacts $F_{j}\left(t\right)$ remaining after physical distancing in county j at time t is therefore

$$F_{j}\left(t\right)=w\left(1-\epsilon C_{j}\left(t\right)\right)+\left(1-w\right)\left(1-\epsilon \left(1-\exp\left(-\omega P_{j}^{+}/P_{j}\right)\right)\right).$$

The contacts of an infected person decline from $f_{T=N}=1$ to a fraction $f_{T=K}=1-\eta$ if they test positive for COVID-19, where η=0.8 (39, 40). The transmission probability also depends on the individual’s epidemiological state D, with $\beta_{D_{ns},j}=\beta^{D_{0},j}$ for nonsuperspreaders $\left\{A_{ns},\cdot \right\},\left\{I_{ns},\cdot \right\}$ and $\beta_{D_{s},j}=\beta^{D_{0},j}\left(1-s\right)/s$ for superspreaders $\left\{A_{s},\cdot \right\},\left\{I_{s},\cdot \right\}$, as well as local differences in transmission. Hence the probability per day that a susceptible person in county j is infected by an infectious person is

$$\lambda_{j}\left(t\right)=1-\underset{D,T}{\prod }{\left[1-F_{j}\left(t\right)f_{T}\beta_{D,j}\left(\xi /\left(1+cP_{j,t}^{*}\right)+\left(1-\xi \right)/P_{j,t}^{*}\right)\right]}^{P_{j,t}^{*DT}},$$

since this is 1 minus the probability of not being infected by any class of the infectious individuals in county j. The starred notation, $P_{j,t}^{*}$ (respectively $P_{j,t}^{*DT}$) denotes the population size (respective number of individuals of disease status D and testing status T) on day t after adjusting for travel. ξ and c control how the transmission probability depends on population size: Standard incidence is recovered for ξ=0, while ξ=1 recovers a scenario where contacts increase with population size to an extent controlled by c (22).

### School and Workplace Closure.

In Ontario, an emergency was declared on the day the cumulative number of reported positive cases $t_{n}$ reached 325 (March 17), leading to closures of workplaces (schools were already closed for March Break). Hence, closure strategies in our model were enacted only after an initial $t_{start}=75$-d province-wide closure expires. Reopenings (and reclosures) are subsequently enacted under the local and global strategy when the percentage of confirmed cases falls below (or exceeds), a trigger prevalence $\gamma_{G}$ at the province level or $\gamma_{l,j}$ within a county j. Under limited coordination, $\gamma_{l,j}$ may be greater in counties eager to lift or hesitant to enact closures. Any closures last for $\delta_{C}=50$ d, with $t_{G}$ ($t_{l,j}$) denoting the last time a closure was enacted in the province (in county j), after which period the closure decision is reevaluated. The closure function $C_{j}\left(t\right)$ is then

$$C_{j}\left(t\right)=\left\{\begin{array}{cc} 1\; & t_{n=325}<t<t_{start}\\ 1\; & t>t_{start}+t_{n=325},\left(P_{G}^{+}/P_{G}>\gamma_{G}\text{or}t<t_{G}+\delta_{C}\right)\\ 1\; & t>t_{start}+t_{n=325},\left(P_{j}^{+}/P_{j}>\gamma_{l,j}\text{or}t<t_{l,j}+\delta_{C}\right)\\ 0\; & \text{otherwise}, \end{array}\right.$$

where $P_{G}^{+}$ is the total number of known, active cases in the province and $P_{G}$ is province population size.

### Calibration.

We set $\beta_{0}^{A}=\beta_{0}^{P}=0.5\beta_{0}^{I}$ (46), based on data showing 44% of SARS-CoV-2 shedding occurs before symptoms develop and our assumed duration of infectious periods. Given that contact rates can depend on population size, we determined $\beta_{0}^{I}$ for each value of ξ and c by calibrating the model in the absence of distancing, closure, and testing such that 65% of the population is infected, based on the assumption that $R_{0}=2.3$ and using final size projections from compartmental epidemic models (19, 47). We allowed for the county-specific baseline transmission probability ${\beta_{0}^{I}}_{j}$ to vary within ±15% of the province-level value $\beta_{0}^{I}$ and ensured the province-wide average of fitted ${\beta_{0}^{I}}_{j}$ values (weighted by population size) equaled $\beta_{0}^{I}$. To avoid overfitting, we constrained $\beta_{0}^{I}=1$ for Toronto (which depends on c) and assumed $\beta_{0}^{I}$ is the same among counties within each county group. We calibrated distancing parameters (ϵ, ω), testing ($\tau_{I}^{t_{0}}$, $\tau_{I}^{t_{f}}$), the dependence of transmission probability on population size (ξ, c), and location j by fitting model outputs of 1) time series of incident confirmed cases (the number of individuals entering the {I,K} state each day) to empirical data on daily confirmed cases by reporting date in each county or county group (26) (*SI Appendix*, Fig. 5); 2) the modeled ratio of actual cases to confirmed positive cases province-wide (i.e., number of individuals not in {S,⋅} to the cumulative number of individuals tested positive), to an empirical estimate of this ratio of 8.76 for underascertainment in the United States (32) (10.2±4.9 in our model, mean±2 SD); and 3) the modeled discretionary distancing $1-\exp\left(-\omega P_{j}^{+}/P_{j}\right)$ by day $t-t_{n=325}=21$ in our simulation to an empirical estimate of ∼50% adherence to physical distancing by day 21 of the outbreak (April 6, 2020) from a public survey (48) (0.57±0.3 in our model). To visualize the spatial case distribution, we calculated the proportion of all confirmed positive tests found in the four most populous Ontario cities and in three sets of counties grouped by population density. We adjusted the proportion of total cases and total cases per 100,000 (Fig. 1*A*) in each county/county group j for spatial differences in number of tests $T_{j}$ per capita by multiplying the observed number of cases in each location by the fraction $\left(P_{j}\sum T_{j}\right)/\left(T_{j}\sum Pj\right)$ (with values ranging from 0.72 to 1.16).

## Supplementary Material

Supplementary File

## Data Availability

All data used in this paper are publicly available (see references). Code and data for model simulation and fitting is available in GitHub (49).
