# Supplementary material for: Local lockdowns outperform global lockdown on the far side of the COVID-19 epidemic curve
Source: Proc Natl Acad Sci U S A. 2020 Sep 4;117(39):24575–80. doi: 10.1073/pnas.2014385117 (PMC7533690; doi:10.1073/pnas.2014385117)
Supplement: Supplementary File [file pnas.2014385117.sapp.pdf]

Supplementary Information for

Local lockdowns outperform global lockdown on the far side of the COVID-19 epidemic curve

Vadim A. Karatayev, Madhur Anand, Chris T. Bauch

Vadim A. Karatayev.
E-mail: vkaratay@uoguelph.ca

This PDF file includes:

Figs. S1 to S5
Table S1
SI References

www.pnas.org/cgi/doi/10.1073/pnas.2014385117

Table S1. Parameter definitions, baseline values and literature sources

Parameter	Meaning	Baseline Value	Source
$\tau_I^{t_0}$	symptomatic testing probability for $t < t_{n=325}$	0.023/day	(1–3), calibrated
$\tau_I^{t_f}$	symptomatic testing probability for $t > 60 + t_{n=325}$	0.46/day	(1–3), calibrated
α	transition probability, $E \rightarrow A$	0.4/day	(4, 5)
σ	transition probability, $A \rightarrow I$	0.4/day	(4, 5)
ρ	transition probability, $I \rightarrow R$	0.67/day	(4, 5)
w	proportion of contacts in schools and workplaces	0.45	(6)
ω	risk perception proportionality constant	5.7 $\times 10^4$	(1–3), calibrated
ϵ	physical distancing efficacy	0.64	(1–3), calibrated
β_0^A	transmission probability, asymptomatic	5.4×10^{-6}/day	(7, 8), one-half β_0^I
β_0^I	transmission probability, symptomatic	1.1×10^{-5}/day	(7, 8), calibrated
$\beta_{0\,j}^I$	City- and county-group-specific transmission probability (constrained $\beta_{0\,Toronto}^I = \beta_0^I$)	Peel: $0.98\beta_0^I$<br>York: $0.88\beta_0^I$<br>Ottawa: $0.93\beta_0^I$<br>250-500: $0.83\beta_0^I$<br>100-250: $1.05\beta_0^I$<br>< 100: $1.15\beta_0^I$	calibrated
ξ	incidence function control parameter	0.2	(9–11), calibrated
c	transmission probability constant	7.0×10^{-6}	calibrated
s	superspreading parameter	0.2	(12)
π	proportion of asymptomatic individuals	0.2	(13)
η	adherence to isolation	0.8	(14, 15)
r	reduced travel rate if symptomatic	0.19	(16)
m_{jk}	connectivity matrix	see Methods	(17)
γ_l	Threshold to close county	varied	
γ_G	Threshold to close province	varied	
δ_C	Minimum closure duration	30 days	

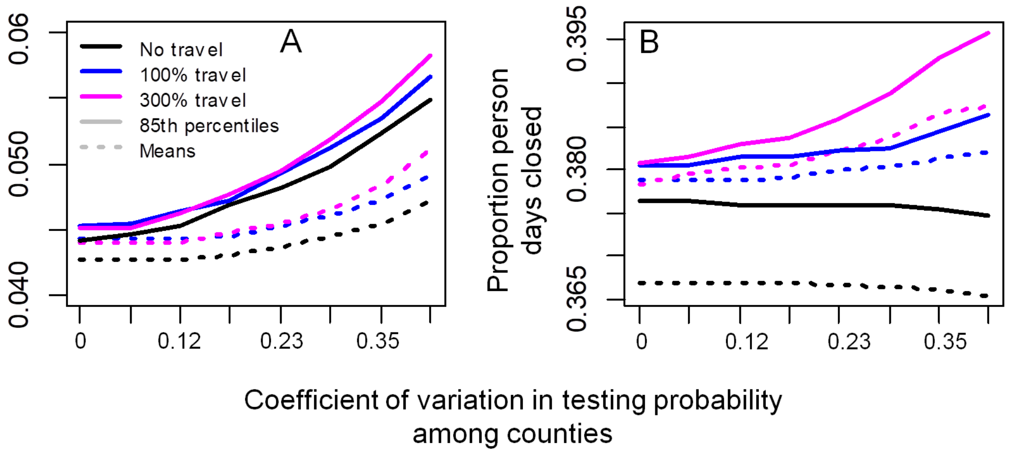

Fig. S1. Decreasing coordination in testing of symptomatic individuals across counties increases total cases and person days closed under the local strategy. $\tau_{I,j}$ expresses uncorrelated variation among counties according to a uniform distribution with mean $\tau_I = 0.46$.

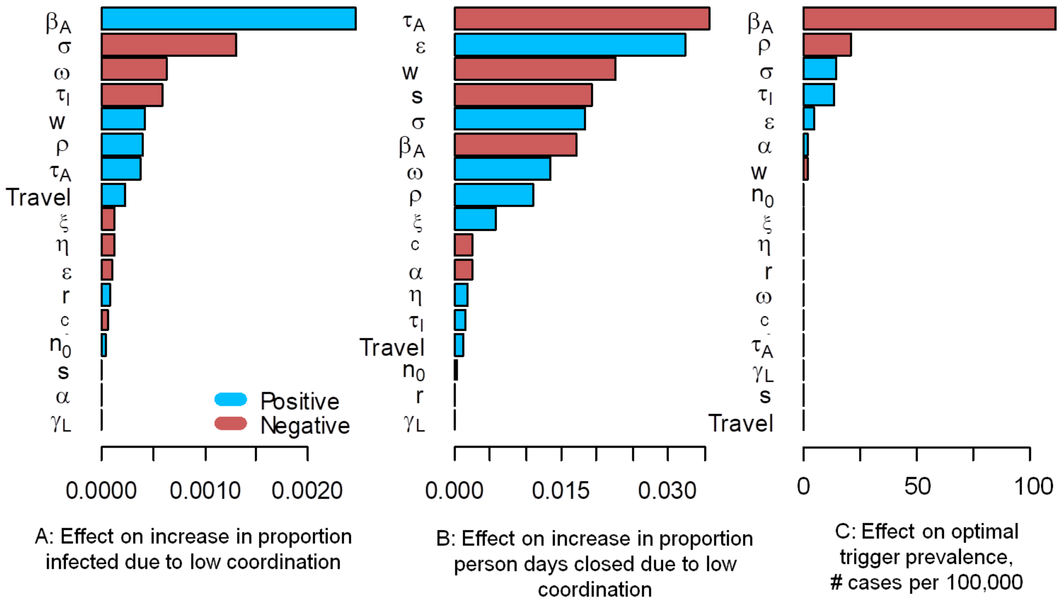

Fig. S2. Effects of $\pm 20\%$ variation in each parameter around its default value (Table 1) on the importance of coordination and the optimal trigger prevalence for the local strategy. Note that disease progression parameters can have counter-intuitive effects because they also regulate the number of positive, active cases on which closures and distancing depend.

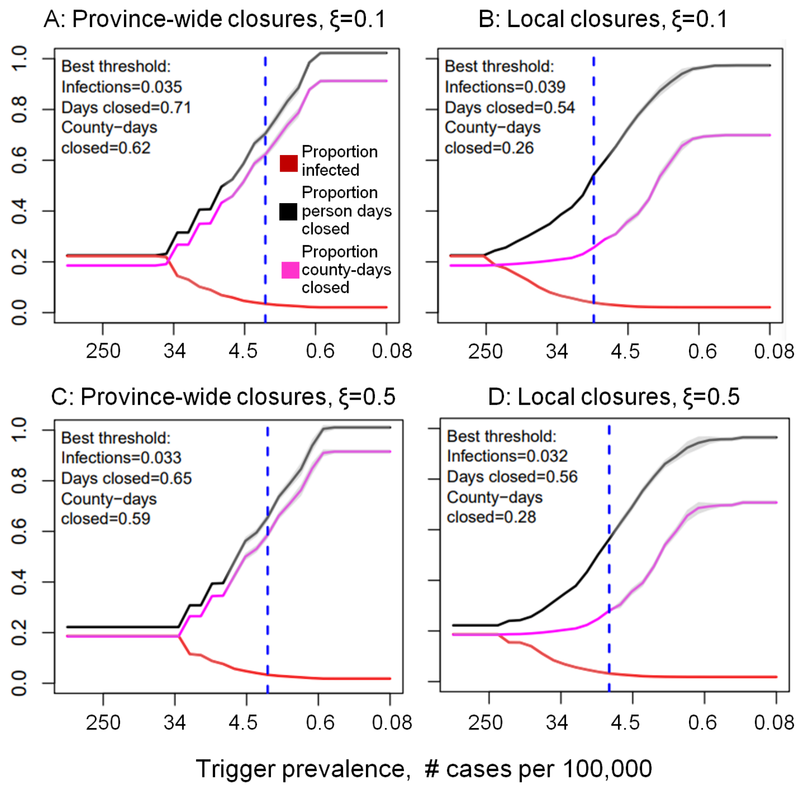

Fig. S3. Comparison of province-wide (A, C) versus county-by-county (B, D) closure strategies as the effect of county population on transmission probability ξ increases from 0.1 (A, B) to 0.5 (C, D). For each ξ value we re-calibrated the model parameters for best fit.

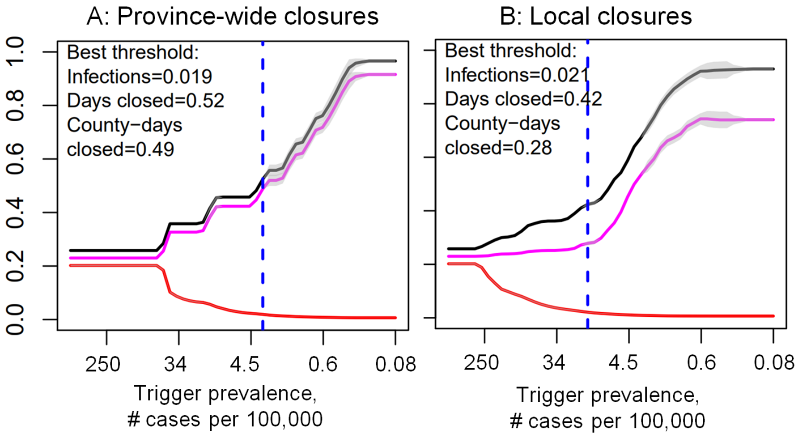

Fig. S4. Comparison of province-wide (a) versus county-by-county (b) closure strategies under 100% higher travel rates compared to Fig. 3 of the main text. Other settings are as in Fig. 3.

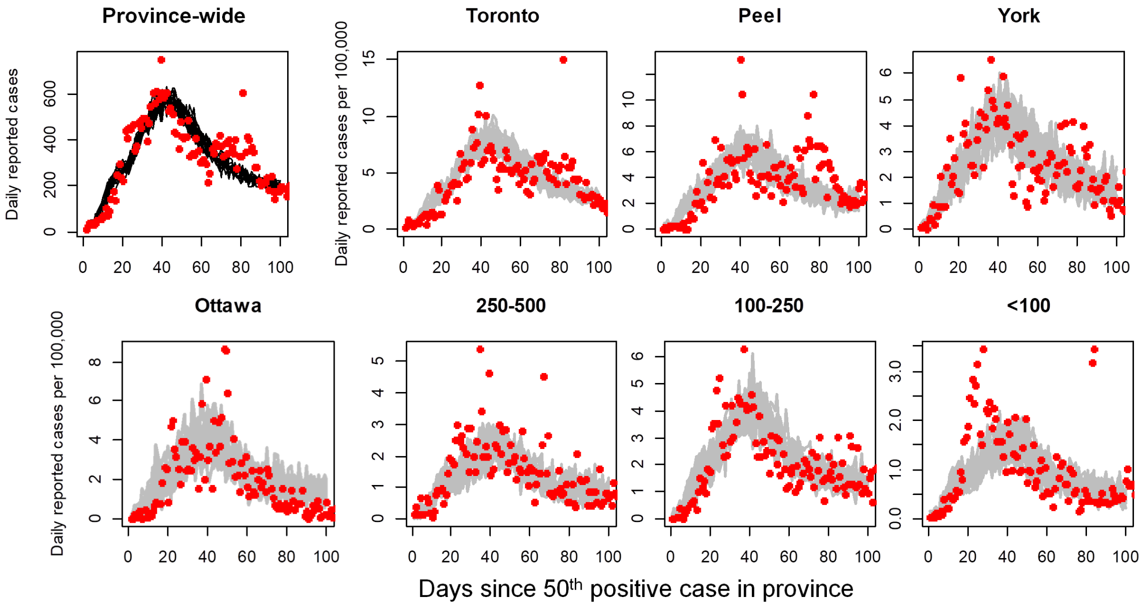

Fig. S5. The fit of 15 stochastic model implementations (black lines) to daily reported positive cases (red dots) province-wide (top-left panel) and to each city or county group (following Fig. 1). The large spikes apparent near the peak of the epidemic curves in the empirical data are due to intense institutional outbreaks and thus are not captured by our population-level model.

References

1. L Saad, Americans step up their social distancing even further. https://news.gallup.com/opinion/gallup/298310/americans-step-social-distancing-even-further.aspx (2020; accessed March 31, 2020).
2. Ontario, Epidemiological summary. covid-19 cases by reported date (figure 1) https://files.ontario.ca/moh-covid-19-report-en-2020-04-06.pdf (2020; accessed March 31, 2020).
3. A Lachmann, Correcting under-reported covid-19 case numbers. *medRxiv* (2020).
4. H Nishiura, NM Linton, AR Akhmetzhanov, Serial interval of novel coronavirus (2019-ncov) infections. *medRxiv* (2020).
5. L Tindale, et al., Transmission interval estimates suggest pre-symptomatic spread of COVID-19. *medRxiv* (2020).
6. Bureau of Labor Statistics, American time use survey — 2018 results. https://www.bls.gov/news.release/pdf/atus.pdf (2018) accessed 4 April 2020.
7. AR Tuite, DN Fisman, AL Greer, Mathematical modelling of covid-19 transmission and mitigation strategies in the population of ontario, canada. *CMAJ* (2020).
8. J Hilton, MJ Keeling, Estimation of country-level basic reproductive ratios for novel coronavirus (covid-19) using synthetic contact matrices. *medRxiv* (2020).
9. J Antonovics, Y Iwasa, MP Hassell, A generalized model of parasitoid, venereal, and vector-based transmission processes. *The Am. Nat.* **145**, 661–675 (1995).
10. H Hu, K Nigmatulina, P Eckhoff, The scaling of contact rates with population density for the infectious disease models. *Math. biosciences* **244**, 125–134 (2013).
11. S Heroy, Metropolitan-scale COVID-19 outbreaks: how similar are they? *arXiv preprint arXiv:2004.01248* (2020).
12. JO Lloyd-Smith, SJ Schreiber, PE Kopp, WM Getz, Superspreading and the effect of individual variation on disease emergence. *Nature* **438**, 355–359 (2005).
13. K Mizumoto, K Kagaya, A Zarebski, G Chowell, Estimating the asymptomatic proportion of coronavirus disease 2019 (covid-19) cases on board the diamond princess cruise ship, yokohama, japan, 2020. *Eurosurveillance* **25**, 2000180 (2020).
14. F Soud, et al., Isolation compliance among university students during a mumps outbreak, kansas 2006. *Epidemiol. & Infect.* **137**, 30–37 (2009).
15. J Hellewell, et al., Feasibility of controlling covid-19 outbreaks by isolation of cases and contacts. *The Lancet Glob. Heal.* (2020).
16. CDC, Severe outcomes among patients with coronavirus disease 2019 (COVID-19)—united states, february 12–march 16, 2020. (2020).
17. Statistics canada, 2016 census, catalogue no. 98-400-x2016391. (Accessed April 7, 2020).
